# Is sagittal sinus resection in falcine meningiomas a factor of bad surgical outcome?

**DOI:** 10.4103/2152-7806.71983

**Published:** 2010-10-25

**Authors:** Paulo Henrique Pires de Aguiar, Rogério Aires, Marcos Vinicius Calfatt Maldaun, Adriana Tahara, Antonio Marcos de Souza Filho, Carlos Alexandre Zicarelli, Ricardo Ramina

**Affiliations:** 1 Department of Neurology - São Paulo Medical School, University of Sao Paulo, São Paulo; Division of Neurosurgery of Santa Paula Hospital, São Paulo; Division of Neurosurgery of Brigadeiro, Unic Brazilian Health System- (SUS-UBHS), São Paulo; Division of Neurosurgery of São Camilo Hospital, São Paulo; Department of Surgery, Catholic Pontific University, Curitiba, Brazil; 2 Division of Neurosurgery of Santa Paula Hospital, São Paulo; Division of Neurosurgery of Brigadeiro, Unic Brazilian Health System- (SUS-UBHS), São Paulo; Division of Neurosurgery of Santa Casa, Londrina, Paraná; Division of Neurosurgery of São Camilo Hospital, São Paulo, Brazil; 3 Division of Neurosurgery of Santa Paula Hospital, São Paulo; Division of Neurosurgery of São Camilo Hospital, São Paulo, Brazil; 4 Division of Neurosurgery of Santa Paula Hospital, São Paulo; Division of Neurosurgery of Brigadeiro, Unic Brazilian Health System- (SUS-UBHS), São Paulo; Division of Neurosurgery of São Camilo Hospital, São Paulo, Brazil; 5 Division of Neurosurgery of Santa Paula Hospital, São Paulo; Division of Neurosurgery of Santa Casa, Londrina, Paraná, Brazil; 6 Department of Surgery, Catholic Pontific University, Curitiba, Brazil

**Keywords:** Falcine meningioma, falx meningiomas – falx cerebri, surgical results

## Abstract

**Objective:**

Meningiomas arising purely from the falx below the longitudinal sinus represents a surgical challenge for the neurosurgeon. The authors discuss the new aspects of surgical details that may avoid complications and determine the prognosis.

**Materials and Methods:**

We retrospectively evaluated our surgical experience from June 2004 to January 2010. Seventy patients harboring falcine meningiomas were included and submitted for surgical resection. All historical records, office charts and images were reviewed in order to sample the most important data regarding epidemiology, clinical pictures, radiological findings and surgical results, as well as the main complications. The patients were divided into three main groups: anterior third 32 patients (Group A), middle third 15 patients (Group B), 23 patients in the posterior third of falx (Group C).

**Results:**

In Group A, total macroscopic resection was achieved in 31 out of 32 cases (96.87%). Twenty five patients had Rankin 0, five patients had Rankin 1-2, two patients had Rankin 6. In Group B (15 patients), 10 patients had gross resection and Rankin 0, four patients had Rankin 1-2 and one patient had Rankin 6. In Group C (23 patients), 20 patients were absolutely able, Rankin score 0, after six months postoperative period (83.3% had excellent results) and no mortality. Four cases had Rankin score 1 – 2 (16.6%). Ten cases (43.47%) had Simpson I resection and ten cases (43.47%) had Simpson II.

**Conclusion:**

Despite larger lesion volumes, Group A meningiomas had a better outcome due to the position they were in, the tumor and surrounding structures. The preoperative preparation and surgical planning can preserve sagittal sinus; but in some cases, this is not possible. Sagittal sinus resection, as proven by this paper, is still a factor of bad surgical outcome. In the middle and posterior third, resection of sagittal sinus is a factor of a bad outcome, due to cerebral infartion.

## INTRODUCTION

The authors presented their experience in falcine meningioma surgery with regard to neurosurgical treatment of 70 falcine meningiomas from June 2004 to January 2010, discussing the main aspects of surgical details that may avoid complications, and determining the prognosis. The treatment of anterior third meningiomas is easier than that of posterior third. This can be explained because of the anatomical localization and neighboring structures.

Meningiomas arising purely from the falx below the longitudinal sinus represent a complex technical problem and also will be discussed at this time.

Falcine meningioma, as defined by Cushing, is a meningioma arising from the falx cerebri and completely concealed by the overlying cortex.[9] However, in practice, many falcine meningiomas involve the sagittal sinus. Falcine meningioma tends to grow predominately into one cerebral hemisphere but is often bilateral, and in some patients the tumor grows into the inferior edge of the sagittal sinus.

The pure falcine meningiomas (without relation with cortical surface and superior sagittal sinus) and parasagittal falcine meningiomas may arise at any point along the midline, anterior to posterior, and present different technical problems depending on their location and depth. Cushing and Eisenhardt[9] defined parasagittal meningioma as one that fills the parasagittal angle with no brain tissue between the tumor and superior sagttal sinus. Sometimes, it invade partially or completely the superior sagttal sinus.

## MATERIALS AND METHODS

Seventy patients harboring on falcine meningiomas and 85 parasagittal meningiomas without attachment to falx were surgically treated between June 2004 and January 2010 by the main author in four different Hospitals in Brazil. In this paper, we exclude the second group of patients without attachment to falx, including only the cases of falcine meningiomas. All historical, clinical radiological and surgical records have been kept in order to be reviewed by the authors for statistical analysis. We classified them into pure falcines (those meningiomas attached to falx, without relationship to cortical surface and superior sagittal sinus) and falcines (predominantly attached to falx, with portion attached to superior sagittal sinus). The average age was 55.4 years old (from 19 to 78 years old) and male to female ratio was 1: 6, and main location: anterior third 32 patients (Group A), middle third 15 patients (Group B), 23 patients in the posterior third of falx (Group C).

The clinical features are presented in Table 1.

**Table 1:** Clinical features

Groups clinical features	Group A	Group B	Group C
Headache	21 patients	7 patients	2 patients
Mental disturbance	10 patients	4 patients	None
Seizure	5 patients	4 patients	2 patients
Focal deficits	2 patients	7 patients	8 patients
Asymptomatic	6 patients	4 patients	2 patients

The hypervascularization was found in 3, 8, and 8 respectively according to the anterior, middle, posterior position on the falx in Groups A, B, and C. The pure falcine (without contact with cortical surface or superior sagittal sinus) was 15, 2, 10, respectively, in the Groups A, B, and C. The angiogram by MRI was performed in 60 out of 70 cases, except for 10 of Group A; however, an angiogram by digital angiography was performed in 20 cases (in all 19 hypervascularized). An embolization was needed in 4 cases of Group B (4 cases in 8 hypervascularized) and in 4 cases of Group C (4 cases in 8 hypervascularized).

In Groups B and C, the Trollard vein was identified in the middle of surgical field making some difficulty in the dissection of a tumor in almost 19 cases (50%), anterior to surgical field in 9 cases, and posterior in 10 cases. All of hypertrophic Trollard vein systems were found in the middle of surgical field, in a total of 9 cases, and 2 hypoplastic Trollard vein systems were found in an anterior position.

None of the cases presented preoperative intra-tumoral bleeding or intraparenchymal hematoma or subdural hematoma.

### Perioperative routine preparation

Anticonvulsants are started in the immediate pre-operative period. They should be given intravenously on the night prior to surgery. Corticosteroids are given routinely the night before surgery[20] (dexametasone 4 mg IV every6 h). On the morning of surgery, steroids are administered again and 10% mannitol (500ml) is given at the time of induction of general anesthesia in the operating room. Pneumatic compression boots should be applied at this time in an effort to avoid postoperative pulmonary emboli.

### Standard surgical technique

**Figure 1:**
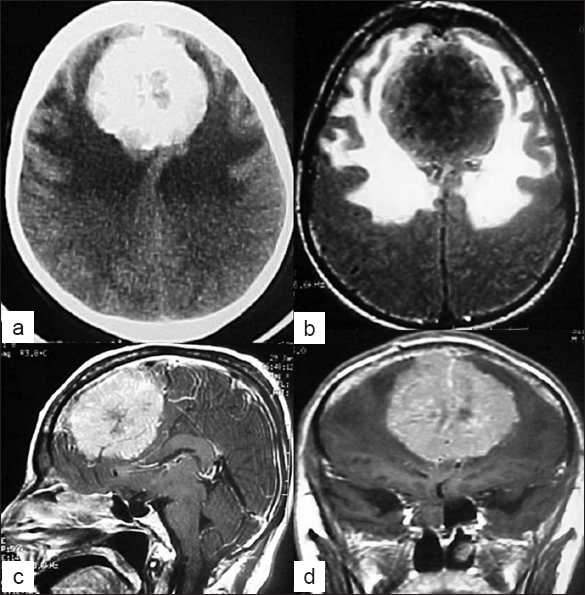
Example of Group A meningiomas – anterior localization – huge meningiomas with perilesional edema in MRI (a,b,c,d)

For anterior falcine meningiomas [Figure 1], the patient is placed in a supine position, with the head in neutral position, fixed in 3 pin Mayfield (Codman, USA) head fix or multiple pin Sugita (Myzuho, Japan) head fix. The operative head field is cleaned with iodine or chlorexidine (alcoholic solution). The incision may be anterior curvilinear shaped or following the coronal suture. The craniotomy is performed with a high speed drill Midas Rex (Meditronic, USA) or Legend (Meditronic, USA) or Anspach, (Anspach, USA), using generally 4 holes of trepanation, including the midline always in the craniotomy in order to access both sides. In a few cases where the meningioma is placed deep, we use the portable neuronavigator Stelth (Meditronic, USA). The dura is opened bilaterally turned to superior sagittal sinus, and if the sinus is occluded we accomplish immediately the closure of sinus with Prolene 2.0 suture (Johnson, USA). The tumor insertion in the falx is visualized under microscopic magnification Pentero or NC 31 (Carl Zeiss, Germany) and a sharp dissection and coagulation is done to obtain complete devascularization in the insertion. The capsule is coagulated and the cortical surface is gently retracted with a Leila spatula (blade) in a Leila retractor (Codman, USA) in order to expose the cleavage planes between the tumor and cortex, pledgets of cotton are placed separating the tumor from the cortex. The tumor is removed on one side first, and the falx implantion is resected, then the other side is covered. The tumoral debulking is accomplished with ultrasonic aspirator Sonopet (Japan) or Sonoca (Germany) and when the tumor has its core removed, we begin to turn in the capsula with forceps and cut it in small pieces until complete resection. The infiltrated falx is cut in complete extension. The hemostasis is performed with Surgicel (Johnson, USA) or Excel (Assute, USA) and bipolar coagulation. The closure is with watertight sutures using dural substitute as Duragen (Integra, USA) or Preclude (Gore, USA). The bone flap is fixed with bone miniplates, type Medartis (Medartis, Swiss). When this technique is used, the chance of problems in this approach is minimized, such as extensive bleeding or cerebral infarction.

**Figure 2:**
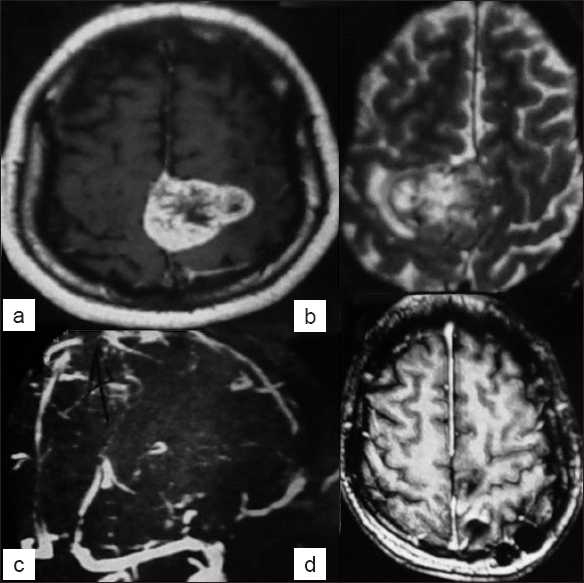
Example of Group B meningiomas – middle localization – large meningioma with perilesional edema in MRI (a,b,d) and commitment of superior sagittal sinus (c)

For middle falcine Meningiomas [Figure 2], the patient is placed in a lateral or supine position, with the head turned to opposite tumor side, fixed in 3 pin Mayfield (Codman, USA) head fix or multiple pin Sugita (Myzuho, Japan) head fix. The incision is normally horseshoe shaped or extensive straight linear shaped, and all other procedures are very similar to the above mentioned, except for the care of the Trollard vein during the dissection, and we always try to avoid closing the sagittal sinus in this position. The burr holes are done on both sides of skull, near the superior sagittal sinus. The sinus has almost never been injured, but when this happens, we try to stop the bleeding using cotton and raising the head. We generally dissect the Trollard vein and protect it with cotton. The vein should be observed and monitored over the entire length of operative field. In this case, the sagittal sinus is left in place. We do not try to remove it, because if we injured the sagittal sinus, edema and cerebral infarction can happen. When edema occurs, craniotomy is the surgical procedure and it is left without bone flap for six months.

**Figure 3:**
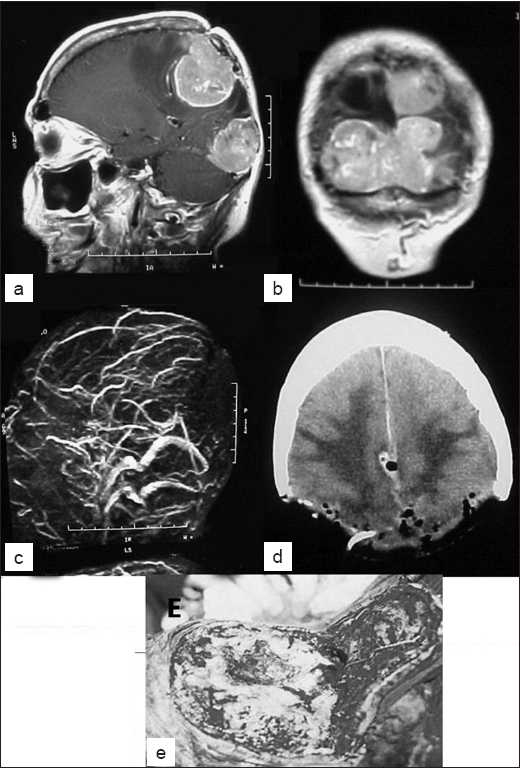
Of Group C meningiomas – posterior localization – multiple lesions reaching posterior fossa and occipital lobe (a,b,c) and Simpson 1 resection (d) and surgical view (e) The arrow shows stenosis in Figure c

For posterior falcine Meningiomas [Figure 3], the patient is positioned at a ¾ prone, with the head turned down, fixed in 3 pin Mayfield (Codman, USA) head fix or multiple pin Sugita (Myzuho, Japan) head fix. The incision is horseshoe shaped, and all other procedures are very similar to the above mentioned, except for the care of straight sinus and Galen vein during the tumor dissection, and we always avoid closing the sagittal sinus in this position, and mainly avoid damage to the torcular venous area. For multiple lesions [Figure 3], we approach the posterior fossa first and then the others. In this specific case, we put the patient in a ¾ prone position, and operate on the fossa posterior with a median incision and a horseshoe incision, the parieto-occipital was approached. The superior sagittal sinus was not resected, although both lesions cross the midline. A craneoplasty was done six months after surgery.

The bone flap is replaced and held firmly in place with interrupted mini plates of titanium. The bone margins and burr holes are filled in with acrylic to provide an excellent cosmetic result, and the wound is closed with galeal sutures and skin staples. A subgaleal drain 3.2mm of internal diameter is brought out through a separate stab incision and is left in place for the first 48 h of the postoperative period. In cases where bony resection is needed, either autologous bone grafts or pre-manufactures prostheses could be further utilized.

In order to avoid venous complications and postoperative edema, we must observe the imperative steps: first, approach the insertion of meningioma in the falx and coagulate and cut all feeders. This maneuver permit diminishing bleeding during the debulking; second, we must separate by means of microsurgical techniques the arachnoid plan between the tumor and brain parenchyma coagulating pial feedings. Slices of cottons must be placed in this space in order to avoid venous pial bleeding; third, the main veins placed at the surface of the tumor must be above identified and microsurgically dissected and gently pushed out of the surgical view and surgical space of work. The vein must be irrigated by saline solution frequently and protected by a slice of cotton. Fourth, the debulking must accomplished under microscopic view and by means of 4mm ultrasonic aspirator. Fifth, after an important debulking, the pseudocapsule must be fold, turned to the central point (centripetal movement) with bipolar; tumor forceps.

Sixth, if the tumor has adherence or invade the sagittal sinus, we must protect the important venous pathway. When the sinus is completely occluded, the total removal of the occluded sinus may be done. If the occlusion is partial, a consideration must be taken regarding the clinical status. The wall of sinus can be reconstructed with dural patch or pericranio but a considerable risk of thrombosis is present. In this case, meningiomas with partial occlusion in middle and posterior third of sagittal sinus are implicated with high risk of complications.

## RESULTS

### Pathological findings

The pathological findings were: benign in 62 cases (88.57%), atypical in 5 cases (7.14%) and malignant in 3 cases (4.28%). The benign tumors presented through routine pathological diagnostic with hematoxilin and eosin and the following variants of meningiomas: meningothelial in 49 cases (79%), transitional in 11 cases (17.74%), fibroblastic in 1 case (1.61%) and microcistic in 1 case (1.61%).

### Pre operatory embolization

The embolization was accomplished in 4 cases of Group B (4 cases in 8 hypervascularized) and in 4 cases of Group C (4 cases in 8 hypervascularized), being effective (helped the surgeon to control the intra operatory bleeding) in all cases of Group C and only 2 cases of Group B. In 2 cases, the embolization did not help to avoid bleeding and in 2 cases of Group B, in which the embolization proved to be effective, there were skin ischemia followed by extensive necrosis, and plastic reconstruction was found to be necessary in 1 case with good functional and esthetic result, and in the other case it was necessary for a multiple plastic reconstruction and treatment for both osteomielitis and cutaneous infection. The final result for this case was fair, and being a patient with atypical meningioma he could not undergo radiotherapy. Summarizing, the embolization proved to be useful in 75% of cases, and non effective in 25%. In 33.3% of the embolized patients with effectiveness to avoid bleeding, there were severe complications of skin necrosis.

Radiotherapy as adjuvant treatment may be used, but we prefer a new approach for this lesion.

### Surgical results

#### Grade of surgical resection [Table 2]

**Table 2:** Grade of resection

*N*=70	Group A *N*=32	Group B *N*=15	Group C *N*=23
Grade of resection			
Simpson I	31 (96.87)	10 (66.66)	10 (43.47)
Simpson II	1 (3.125)	4 (26.66)	10 (43.47)
Simpson III	0 (0)	1 (6.66)	3 (13.04)
Morbidity	5 (6.25)	4 (26.66)	
Mortality	2 (15.62)	1 (6.66)	

Figures in parenthesis are in percentage

#### Group A

Total macroscopic resection was achieved in 31 out of 32 cases (96.87%), Simpson I grade, independent of age or volume. In these 31 cases, the infiltrated falx was cut and removed. Simpson II was gotten in 1 case (3.125%). The resection of superior sagittal sinus infiltrated was removed in 15 cases out of 31 with Simpson I grade resection (7 partial infiltration, 8 sagittal sinus totally occluded by the tumor) 46.87%. One case presented post operative intraventricular bleeding, and was reoperated, and evaluated asymptomatically. We used to do Valsalva maneuver in all cases. This patient presented with no blood in operative field, but during CT scan after surgery, there was a huge clot in operative field.

#### Group B

Total macroscopic resection was achieved in 10 out of 15 cases (66.66%), Simpson I grade, independent of age or volume. Simpson II was gotten in 4 cases (26.66%) and Simpson III in 1 case due to partial infiltration of sinus and falx (6.66%). The complete occluded sagittal sinus by the tumor was removed in 3 cases.

#### Group C

Total macroscopic resection was achieved in 10 out of 23 cases (43.47%), Simpson I grade, independent of age or volume. Simpson II was gotten in 10 cases (43.47% and Simpson III in 3 cases due to partial infiltration of torcular area, straight sinus and Galen vein respectively (13.04%). The complete occluded sagittal sinus by the tumor was removed in 9 cases out of 13 non pure falcine meningiomas in posterior third of falx and 1 case out of 10 pure falcine meningiomas in posterior third of falx. We did not try to remove the partially occluded superior sagittal sinus in any 2 cases of non pure falcine meningiomas.

### Neurological morbidity and mortality

#### Group A

Group A presented 25 patients absolutely able, Rankin Score 0, after six months post operative period (78.12% of excellent results). 5 cases of Rankin Score 1-2 in the same period (15.62% of good results), there were 2 cases of mortality (6.25%). The 2 cases of mortality were older patients with grade of resection Simpson I with resection of invaded superior sagittal sinus, with arterial hypertension, diabetes mellitus and one of them had prostate cancer and no problems during surgery were detected. The 5 cases of mild morbidity were also associated to Simpson I resection, and a removal of superior sagittal sinus was performed.

#### Group B

Group B presented 10 patients absolutely able, Rankin Score 0, after six months post operative period (66.66% of excellent results). Four cases of Rankin Score 1-2 in the same period (26.66% of good results), and one case of mortality (6.66%). The mortality case was a patient admitted in the emergency room due to sudden increase in intracranial pressure caused by transtentorial herniation. The case of mortality was an older patient with grade of resection Simpson I with resection of invaded superior sagittal sinus. The 4 cases of mild morbidity were also associated to Simpson I resection and Simpson II in 2 cases, and a removal of superior sagittal sinus was performed in 1 case of Simpson I and 1 case of Simpson II resection and no venous infarction was detected intra or postoperative treatment..

#### Group C

Group C presented with 20 patients absolutely able, Rankin score 0, after six months postoperative period (83.3% of excellent results) and no mortality. Four cases of Rankin score 1 - 2 (16.6%). Ten cases (43.47%) had Simpson I resection and ten cases had (43.47%) Simpson II.

## DISCUSSION

Falcine meningioma is responsible for 8.5% of intracranial meningiomas and the transitional meningioma was the most common subtype of falcine meningioma.[7] The patients with falcine meningiomas with reference to gender had the following ratio of male:female of 1:2.1 and an average age of 55 years. Our series presented 1:6 (men:women) relationship, and the mean age was 55.4 years old.

There are several surgical strategies involved in the treatment of falcine meningiomas and those must be individual and focused to the imaging exams, anatomical data and preoperative status. We intend that a successful result is based on these terms, and they are showed in our results.

The falcine meningiomas arising anterior to the coronal suture are the simplest to handle, because whether they are on the dominant or the non dominant side, they are compromising relatively silent areas of the brain and are situated in a region where the longitudinal sinus can be sacrificed if necessary in order to achieve their total removal. These tumors may be present as mass lesions with progressive organic mental syndromes and can be accompanied by generalized seizures, and sometimes with speech arrest,[27] cognitive impairment such as amnesia in patients with frontal falx meningiomas[23] and Weber syndrome has been also described in patients harboring falx meningiomas.[30] Although all efforts must be done to preserve superior sagittal sinus, when the tumor invade this structure, we decide to resect.

Those falcine meningiomas arising in the middle third of the cranial vault present a more difficult technical problem. Those located on the non dominant side usually compromise the sensory-motor strip at their posterior margin, whereas those on the dominant side involve the supplementary motor area as well.[6] Large tumors in the middle third of the cranial vault can be present with a progressive hemi paresis as well as organic mental syndrome and seizures that are often focal in the onset.[17] Our series show 7 cases of headache and 7 cases of deficit. In addition, with lesions on the dominant side, the seizures may be heralded by speech arrest, a syndrome that derives from a compromise to the dominant supplementary motor area. They may be present as calcified mass attached to falx, being difficult to access without any damage to brain parenchyma.[17]

Finally, falcine meningiomas arising in the posterior third of the cranial cavity, generally posterior to the sensory-motor strip, may produce focal motor seizures and/or seizures arising from compromise to the visual cortex. If located sufficiently far posteriorly, they may involve the junction of the sagittal sinus with the transverse sinus and adjacent tentorium. In depth those tumors may evolve the incisural notch laterally or posteriorly and attach to the tentorium as a true falcotentorial meningiomas, and the removal of these tumors certainly represent a more complex and technical challenge.

The falcine meningiomas may be present with bleeding as intraparenchymal hematomas, subdural hematomas and subarachnoid hemorrhage, causing a clinical finding of apoplexy in the patients.[4,19,22] Hemorrhages occurring in asymptomatic falcine meningiomas are known beforehand to have been described after the internal use of low-dose aspirin for prolonged period.[19] We did not have any hemorrhage presentation of falcine meningiomas.

The most important complication was related to an inadvertent damage to the venous system, leading to a venous infarction in elderly patients.

Correspondingly, surgical compromise of the venous, sinuses and cortical structures become progressively more prohibitive unless the sinuses have been totally occluded by meningiomas invasion or huge meningiomas create a surgical approach in depth by the enlargement of the occupied space, in which case excision becomes somewhat less of a problem.

Gross total resection of the tumor was the single most important predictor of an improved surgical outcome.[7] But we agree that most of our complications could be avoided with a less aggressive treatment.

There are several tumors that grow near the falx and may mimic the falcine meningiomas. Osteochondromas, chondrosarcomas, solitary fibrous tumor of the meninges, epidermoid tumors and metastasis are the most frequent.[10,16,25,29]

Computerized tomography (CT) with and without contrast is the first step in the evaluation of intracranial meningiomas. MRI with and without gadolinium helps better to delineate the tumor in relation to the dural sinus, the tumor interface with the cerebral cortex, presence of significant blood supply, and presence of cysts or other intra-tumoral structures that will add to the complexity and malignant potential of the tumor. Good pre-operative evaluation of falcine meningiomas is also important when integrated with neuronavigation protocols to be utilized in the operating room. Furthermore, the junction between tumor and adjacent brain suggests the presence or absence of an accessible arachnoid plane and enables the surgeon to predict the potential degree of neurologic deficit that may follow surgical removal. Gadolinium-enhanced MRI allows demonstration of tumoral or adjancent dural enhancement. The radiological appearance affords a valid predictor of the degree of dural involvement in the region of the sinus and adjacent falx. This may suggest the presence of syncytium of meningeal cells spreading along the falx from the site of major dural attachment.[11,17,24] All meningiomas were diagnosis more extensive studied with magnetic resonance images.

There are many materials utilized for meningioma embolization.[5,12,13,26] Despite advances in neuroangiographic techniques, more flexible microcatheters and highly responsive microwires, the ultimate goal of pre-operative embolization should be the safe reduction in tumoral vascular supply with the least amount of damage prior to surgical resection. Major complications associated with pre-operative embolization of intracranial meningiomas include access site hematomas (generally the groin area), carotid artery dissection, middle meningeal artery dissection and/or perforation with development of subdural hematoma, and strokes due to inadvertent embolization of collateral supply arteries to the internal carotid or vertebral arterial systems.[13,15,26] In our patients, angiography and embolization were generally done on the day or two days before surgery. The meningiomas’ vascularization was assessed by the internal and external carotid arteries and/or vertebral arteries. Ninety percent was embolized by polyvinyl alcohol particles (150-250*µ*m). Although embolization can reduce hemorrhage in operative field, we indicate this procedure (with embolization team) only when the arterial supply by the internal carotid artery is substantial and benefits are low. When arterial supply is done by external carotid branches, we embolize those hypervascular Meningiomas with hypertrophic feeders.

In the treatment of tumors anterior to the coronal suture, the surgeon can be relatively aggressive with excision of the lateral walls of the longitudinal sinus and compromised falx because the whole dural venous sinus can be ligated in this area, with minimal neurological complications, even if it is patent. If the entire sinus is occluded and the falx is infiltrated, then an incision is developed in the dura on the contralateral side and the involved sinus and subadjacent infiltrated falx are excised in one segment. In such situations, the anterior and posterior aspects of the sinus are closed with stay sutures, passed through the falx beneath the inferior corner of the sinus, before excision of the sinus and residual meningioma. In treating lesions posterior to the coronal suture, the patent longitudinal sinus cannot be removed safely in the fashion just described and excision of lateral wall infiltrated with falcine parasagittal meningioma is difficult and complex. In the past, the lateral sinus wall was sutured successively as the tumor was excised.

According to Barajas *et al*,[1] 2009, large falcine meningiomas may be successfully removed following contralateral interhemispheric approach, providing an excellent example of one approach to directly dealing with large, deep interhemispheric feeding vessels unsuitable for embolization.

For tumors placed posteriorly and in the inferior portion of falx depth, an occipital interhemispheric surgical approach should be tailored to the dural origin and the extent of the tumor as depicted from preoperative MRI. Preservation of the straight sinus and Galenic venous system is always recommended.[2] In those cases, additional resection of the falx and/or incision of the tentorium may be performed with complete resection (Simpson grade 1 and 2) in almost 85% of patients.[2]

Antibiotics are given intraoperatively and for 48 h after surgery. Anticonvulsivants are continued for at least 12 months during the postoperative period or for a longer period if the tumor was placed close to the sensory-motor area and/or if the patient had preoperative seizures.

We must be aware that huge meningiomas, mainly in falx and skull base, may cause intraoperative acute brain swelling as well as significant blood loss. Because of this, we should carefully consider the indication and select proper candidates for presurgical cerebral angiography and tumor embolization due to the inherent risk that is apt to be underestimated.[28]

Post operative bleeding may be observed as a post operatory complication and should be promptly treated. In one case, postoperative bleeding was detected in Group A and promptly treated. This patient had a good recovery after surgery.

During falcine meningioma surgery, we must pay attention to cardiac monitoring due to the risk that the handling of falx and tentorium could provoke cardiac asystole. The mechanical stimulation of the falcine area may result in the hyperactivity of the trigeminal ganglion, thereby triggering TCR.[3] The dorsal region of the spinal trigeminal tract includes neurons from hypoglossal and vagus nerves, and projections have been seen between the vagus and trigeminal nuclei.[3] The vagus provides parasympathetic innervation to the heart, vascular smooth muscle, and abdominal viscera.

The rate of recurrence of falx meningiomas significantly increases in cases of non-radical resection of tumor. Aggressive surgical treatment obviously may present several hazards and may carry an increased risk of unsatisfactory outcome; however, the risk of recurrence is significantly decreased.[21]

Nowak and Marchel,[21] 2007 studied a series of 87 consecutive patients surgically treated for parasagittal and falcine meningiomas and concluded that there were no tumor recurrences following radical resection of the tumor and invaded part of sinus, but two postoperative deaths due to hemodynamic complications were observed. In the other 12 patients, meningiomas were removed but sinus infiltration was left in place; the postoperative period was uneventful but the rate of clinically important re-growth in this group of patients was 25% in long-term follow-up.[21]

Huge sized falcine meningiomas (12.9% of all falcine meningiomas) generally may affect the extent of removal, recurrence rate, postoperative outcome, operative morbidity and mortality rates, and survival time negatively.[31] According to Li *et al*, 2005, the main treatment of meningiomas is surgery; however in some cases the radio-surgery might be indicated. We consider that gamma knife treatment is especially valuable for those patients who are at high risk for surgical complications due to other medical conditions, recurrent malignant meningiomas, in patients who refused to be surgically treated due to risks or those whose lesion is situated in an inaccessible or functionally critical area of the brain, making a surgical approach difficult, but in our series, it was used only for adjuvant purpose.

Linac radiosurgery (LRS) for each disease seems to be a safe and effective treatment when well indicated. However, once serious radiation injuries occur there is no effective therapy, so it is important to make appropriate selection of patients with meningiomas for radiosurgery.[18]

The CyberKnife; (Accuray, Inc., Sunnyvale, CA) may be very useful in the treatment of meningiomas, mainly in patients who could not be treated by single-session radiosurgical techniques.[8] The procedure has proved to be safe. Clinical improvement seems to be more frequently observed with the CyberKnife than in our previous linear accelerator experience.[8]

The treatment of falcine meningiomas using Gamma knife unit has presented similar results to that of radiosurgery and CyberKnife.

The use of a small dural window exposure a strategy that may guarantee the safe total removal of huge intracranial meningiomas avoiding the risk of acute intraoperative brain swelling and herniation.[14]
